# (1*S*,3′*S*,3a′*R*,6′*S*)-6′-(2-Chloro­phen­yl)-3′-[(2*R*,3*S*)-1-(4-meth­oxy­phen­yl)-4-oxo-3-phenyl­azetidin-2-yl]-2-oxo-3′,3′a,4′,6′-tetra­hydro-2*H*,2′*H*-spiro­[ace­naphthyl­ene-1,1′-pyrrolo­[1,2-*c*][1,3]thia­zole]-2′,2′-dicarbo­nitrile

**DOI:** 10.1107/S1600536813009276

**Published:** 2013-04-17

**Authors:** Seenivasan Karthiga Devi, Thothadri Srinivasan, Raju Rajesh, Raghavachary Raghunathan, Devadasan Velmurugan

**Affiliations:** a Centre of Advanced Study in Crystallography and Biophysics, University of Madras, Guindy Campus, Chennai 600 025, India; b Department of Organic Chemistry, University of Madras, Guindy Campus, Chennai 600 025, India

## Abstract

The mol­ecular conformation of the title compound, C_41_H_29_ClN_4_O_3_S, is stabilized by intra­molecular C—H⋯O and C—H⋯Cl hydrogen bonds. The thia­zole ring adopts an envelope conformation with the N atom as the flap, while the pyrrolidine ring has a twisted conformation on the N—C bond involving the spiro C atom. The β la­ctam ring makes dihedral angles of 39.74 (15) and 16.21 (16)° with the mean planes of the thia­zole and pyrrolidine rings, respectively. The thia­zole ring mean plane makes dihedral angles of 23.79 (13) and 70.88 (13) ° with the pyrrolidine and cyclo­pentane rings, respectively, while the pyrrolidine ring makes a dihedral angle of 85.63 (13)° with the cyclo­pentane ring. The O atom attached to the β la­ctam ring deviates from its mean plane by 0.040 (2) Å, while the O atom attached to the cyclo­pentane ring deviates from its mean plane by 0.132 (2) Å. In the crystal, mol­ecules are linked by C—H⋯O hydrogen bonds, forming chains along [010], and C—H⋯π and π-π inter­actions [centroid-centroid distance = 3.6928 (17) Å].

## Related literature

For general background to β-la­ctams, see: Banik & Becker (2000 ▶); Brakhage (1998 ▶). For a related structure, see: Sundaramoorthy *et al.* (2012 ▶).
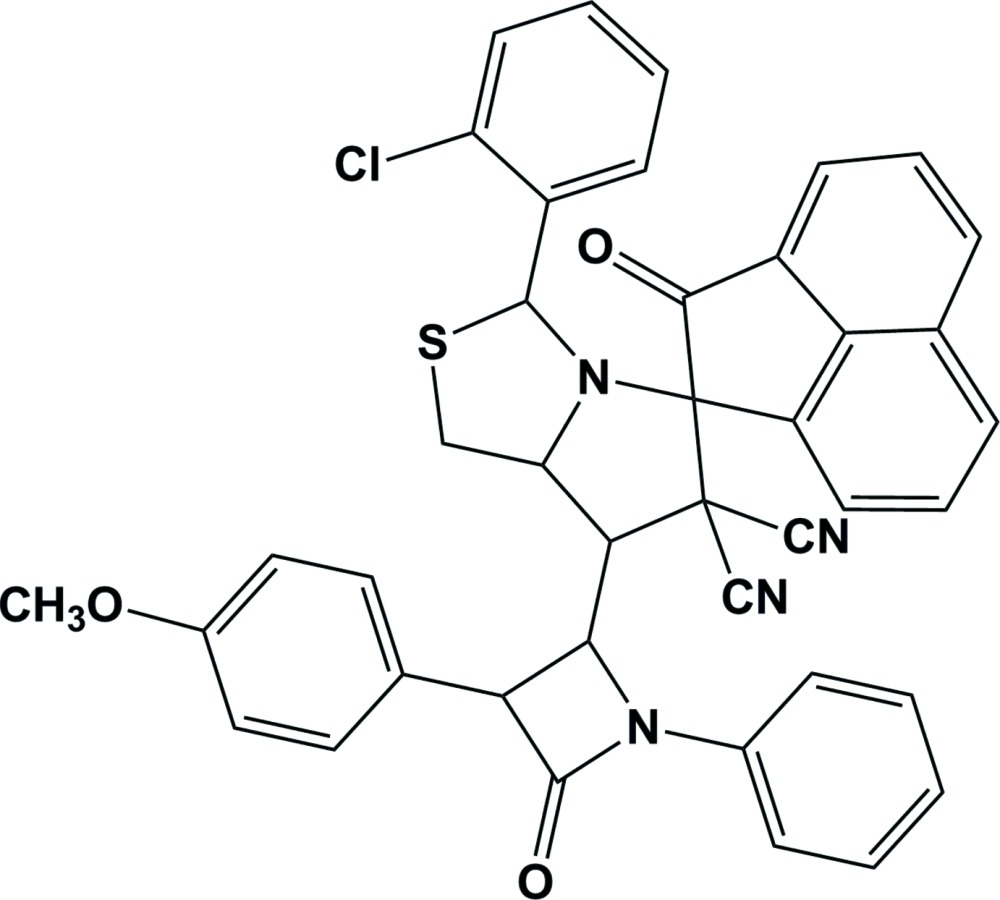


## Experimental

### 

#### Crystal data

C_41_H_29_ClN_4_O_3_S
*M* *_r_* = 693.19
Monoclinic, 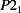

*a* = 10.7611 (5) Å
*b* = 14.3742 (7) Å
*c* = 11.6657 (6) Å
β = 110.107 (3)°
*V* = 1694.50 (14) Å^3^
*Z* = 2
Mo *K*α radiation
μ = 0.22 mm^−1^
*T* = 293 K
0.30 × 0.25 × 0.20 mm

#### Data collection

Bruker SMART APEXII area-detector diffractometer
Absorption correction: multi-scan (*SADABS*; Bruker, 2008 ▶) *T*_min_ = 0.937, *T*_max_ = 0.957
15747 measured reflections
7910 independent reflections
5429 reflections with *I* > 2σ(*I*)
*R*_int_ = 0.034

#### Refinement

*R*[*F* ^2^ > 2σ(*F* ^2^)] = 0.045
*wR*(*F* ^2^) = 0.114
*S* = 1.00
7910 reflections
452 parameters
1 restraint
H-atom parameters constrained
Δρ_max_ = 0.24 e Å^−3^
Δρ_min_ = −0.28 e Å^−3^
Absolute structure: Flack (1983 ▶), 3527 Friedel pairs
Flack parameter: −0.05 (5)

### 

Data collection: *APEX2* (Bruker, 2008 ▶); cell refinement: *SAINT* (Bruker, 2008 ▶); data reduction: *SAINT*; program(s) used to solve structure: *SHELXS97* (Sheldrick, 2008 ▶); program(s) used to refine structure: *SHELXL97* (Sheldrick, 2008 ▶); molecular graphics: *ORTEP-3 for Windows* (Farrugia, 2012 ▶); software used to prepare material for publication: *SHELXL97* and *PLATON* (Spek, 2009 ▶).

## Supplementary Material

Crystal structure: contains datablock(s) global, I. DOI: 10.1107/S1600536813009276/su2576sup1.cif

Structure factors: contains datablock(s) I. DOI: 10.1107/S1600536813009276/su2576Isup2.hkl

Additional supplementary materials:  crystallographic information; 3D view; checkCIF report

## Figures and Tables

**Table 1:** Hydrogen-bond geometry (Å, °) *Cg*1 is the centroid of the C10–C15 ring.

*D*—H⋯*A*	*D*—H	H⋯*A*	*D*⋯*A*	*D*—H⋯*A*
C18—H18⋯O3	0.98	2.36	2.961 (2)	119
C20—H20⋯Cl1	0.98	2.54	3.095 (2)	116
C19—H19*A*⋯O1^i^	0.97	2.54	3.467 (3)	159
C35—H35⋯*Cg*1^ii^	0.93	2.83	3.523 (4)	133

Symmetry codes: (i) 
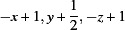
; (ii) 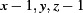.

**Fig. 1.**
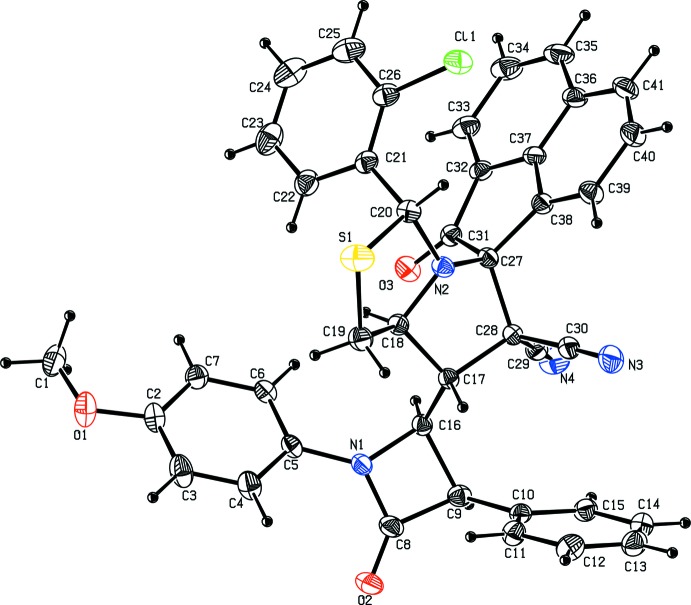
The molecular structure of the title compound, with the atom labelling. Displacement ellipsoids are drawn at the 30% probability level.

**Fig. 2.**
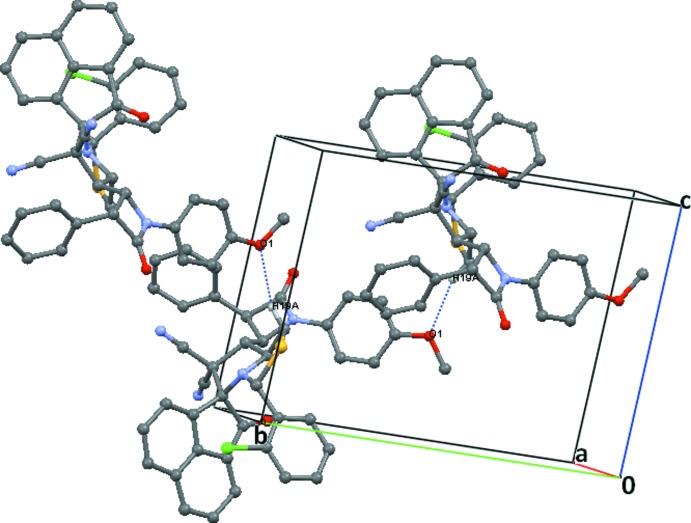
The crystal packing of the title compound viewed along the a axis, showing the hydrogen bonds as dashed lines [see Table 1 for details; H-atoms not involved in hydrogen bonds have been excluded for clarity].

## supplementary crystallographic information

### Comment

The role of β-lactam antibiotics is well known (Banik & Becker, 2000). The most commonly used β-lactam antibiotics for the therapy of infectious diseases are penicillin and cephalosporin (Brakhage, 1998). In view of the potential applications of such compounds we have synthesized the title β-lactam derivative and report herein on its crystal structure.

In the title compound, Fig. 1, the thiazole ring (S1/N2/C18-C20) adopts an envelope conformation with atom N2 as the flap. The pyrrolidine ring (N2/C17/C18/C27/C28) adopts a twisted conformation on bond N2-C27. The β lactam ring (N1/C8/C9/C16) makes a dihedral angle of 39.74 (15)° with the thiazole ring mean plane, and a dihedral angle of 16.21 (16)° with the the pyrrolidine ring mean plane. The β lactam ring makes dihedral angles of 36.83 (16)° and 72.99 (16)° with the methoxy phenyl ring and unsubstituted phenyl ring, respectively. The thiazole ring mean plane makes a dihedral angle of 23.79 (13)° with the pyrrolidine ring mean plane, and a dihedral angle of 70.88 (13)° with the cyclopentane ring (C27/C31/C32/C37/C38) of the acenaphthylen-1(2H)-one ring system. The pyrrolidine ring mean plane is almost normal to the cyclopentane ring with a dihedral angle of 85.62 (13) °. Atom O2 deviates by 0.040 (2) Å from the β lactam ring and O3 deviates by 0.132 (2) Å from the cyclopentane ring. The chlorine atom Cl1 deviates by -0.0427 (8) Å from the phenyl ring to which it is attached.

In the crystal, molecules are linked by C—H···O hydrogen bonds, forming chains along the direction, and C-H···π and π-π interactions [Cg2-Cg3^i^ = 3.6928 (17) Å; Cg2 and Cg3 are the centroids of rings C2-C6 and C32-C37, respectively; symmetry code: -x+1, y-1/2, -z]; see Table 1 and Fig. 2 for details.

### Experimental

A mixture of 2-(2-chlorophenyl)thiazolidine-4-carboxylic acid (1.1 mmol), acenapthenequinone (1.0 mmol) and 2-((1-(4-methoxycyclohexa-2,4-dienyl)-4- oxo-3-phenylazetidin-2-yl)methylene)malononitrile (1.0 mmol) was refluxed in methanol. Completion of the reaction was controlled by TLC. The solvent was removed in vacuo, then the crude product was diluted with chloroform and washed with water followed by brine solution. The organic layer was separated and dried over Na_2_SO_4_. The solvent was removed and the crude product was subjected to column chromatography using petroleum ether: ethyl acetate mixture (7:3) as eluent. The pure product was dried, dissolved in ethyl acetate and heated for two minutes. The resulting solution was subjected to crystallization by slow evaporation of the solvent for 48 hours giving colourless block-like crystals.

### Refinement

The atom coordinates correspond to the absolute structure of the molecule in the crystal [Falck parameter = 0.05 (5)]. The H atoms were placed in calculated positions and refined as riding: C—H = 0.93 - 0.97 Å with U_iso_(H) = 1.5U_eq_(C-methyl) and = 1.2U_eq_(C) for other H atoms.

### Crystal data

**Table:**

C_41_H_29_ClN_4_O_3_S	*F*(000) = 720
*M**_r_* = 693.19	*D*_x_ = 1.359 Mg m^−^^3^
Monoclinic, *P*2_1_	Mo *K*α radiation, λ = 0.71073 Å
Hall symbol: P 2yb	Cell parameters from 7910 reflections
*a* = 10.7611 (5) Å	θ = 1.9–28.4°
*b* = 14.3742 (7) Å	µ = 0.22 mm^−^^1^
*c* = 11.6657 (6) Å	*T* = 293 K
β = 110.107 (3)°	Block, colourless
*V* = 1694.50 (14) Å^3^	0.30 × 0.25 × 0.20 mm
*Z* = 2	

### Data collection

**Table:**

Bruker SMART APEXII area-detector diffractometer	7910 independent reflections
Radiation source: fine-focus sealed tube	5429 reflections with *I* > 2σ(*I*)
Graphite monochromator	*R*_int_ = 0.034
ω and φ scans	θ_max_ = 28.4°, θ_min_ = 1.9°
Absorption correction: multi-scan (*SADABS*; Bruker, 2008)	*h* = −14→14
*T*_min_ = 0.937, *T*_max_ = 0.957	*k* = −18→18
15747 measured reflections	*l* = −15→15

### Refinement

**Table:**

Refinement on *F*^2^	Secondary atom site location: difference Fourier map
Least-squares matrix: full	Hydrogen site location: inferred from neighbouring sites
*R*[*F*^2^ > 2σ(*F*^2^)] = 0.045	H-atom parameters constrained
*wR*(*F*^2^) = 0.114	*w* = 1/[σ^2^(*F*_o_^2^) + (0.0567*P*)^2^] where *P* = (*F*_o_^2^ + 2*F*_c_^2^)/3
*S* = 1.00	(Δ/σ)_max_ = 0.001
7910 reflections	Δρ_max_ = 0.24 e Å^−^^3^
452 parameters	Δρ_min_ = −0.28 e Å^−^^3^
1 restraint	Absolute structure: Flack (1983), 3527 Friedel pairs
Primary atom site location: structure-invariant direct methods	Flack parameter: −0.05 (5)

### Special details

**Table:**

Geometry. All esds (except the esd in the dihedral angle between two l.s. planes) are estimated using the full covariance matrix. The cell esds are taken into account individually in the estimation of esds in distances, angles and torsion angles; correlations between esds in cell parameters are only used when they are defined by crystal symmetry. An approximate (isotropic) treatment of cell esds is used for estimating esds involving l.s. planes.
Refinement. Refinement of F^2^ against ALL reflections. The weighted R-factor wR and goodness of fit S are based on F^2^, conventional R-factors R are based on F, with F set to zero for negative F^2^. The threshold expression of F^2^ > 2sigma(F^2^) is used only for calculating R-factors(gt) etc. and is not relevant to the choice of reflections for refinement. R-factors based on F^2^ are statistically about twice as large as those based on F, and R- factors based on ALL data will be even larger.

### Fractional atomic coordinates and isotropic or equivalent isotropic displacement parameters (Å^2^)

**Table:**

	*x*	*y*	*z*	*U*_iso_*/*U*_eq_	
C1	0.4023 (3)	0.4825 (2)	0.2806 (3)	0.0781 (9)	
H1A	0.3223	0.5111	0.2281	0.117*	
H1B	0.3826	0.4217	0.3037	0.117*	
H1C	0.4642	0.4774	0.2382	0.117*	
C2	0.5027 (3)	0.62498 (17)	0.3725 (3)	0.0566 (7)	
C3	0.5693 (4)	0.6716 (2)	0.4795 (3)	0.0721 (9)	
H3	0.5779	0.6444	0.5542	0.087*	
C4	0.6227 (3)	0.75768 (19)	0.4766 (3)	0.0622 (7)	
H4	0.6672	0.7888	0.5490	0.075*	
C5	0.6102 (2)	0.79803 (15)	0.3656 (2)	0.0455 (6)	
C6	0.5403 (2)	0.75252 (16)	0.2583 (2)	0.0474 (6)	
H6	0.5299	0.7804	0.1836	0.057*	
C7	0.4860 (2)	0.66590 (17)	0.2615 (3)	0.0518 (6)	
H7	0.4385	0.6355	0.1894	0.062*	
C8	0.7964 (2)	0.91484 (18)	0.4313 (2)	0.0551 (7)	
C9	0.7905 (2)	0.99648 (17)	0.3462 (2)	0.0480 (6)	
H9	0.8518	0.9869	0.3017	0.058*	
C10	0.8058 (2)	1.09252 (16)	0.4000 (2)	0.0439 (5)	
C11	0.7753 (2)	1.11136 (19)	0.5039 (2)	0.0542 (6)	
H11	0.7471	1.0635	0.5426	0.065*	
C12	0.7865 (3)	1.2002 (2)	0.5502 (3)	0.0655 (8)	
H12	0.7647	1.2121	0.6193	0.079*	
C13	0.8297 (3)	1.2713 (2)	0.4947 (3)	0.0653 (8)	
H13	0.8367	1.3313	0.5260	0.078*	
C14	0.8622 (3)	1.2538 (2)	0.3938 (3)	0.0590 (7)	
H14	0.8926	1.3018	0.3569	0.071*	
C15	0.8504 (2)	1.16505 (18)	0.3459 (2)	0.0514 (6)	
H15	0.8725	1.1538	0.2768	0.062*	
C16	0.6481 (2)	0.95750 (16)	0.2704 (2)	0.0406 (5)	
H16	0.6442	0.9320	0.1913	0.049*	
C17	0.5310 (2)	1.01889 (15)	0.2601 (2)	0.0391 (5)	
H17	0.5496	1.0516	0.3380	0.047*	
C18	0.3964 (2)	0.96917 (16)	0.2303 (2)	0.0416 (5)	
H18	0.3954	0.9124	0.1834	0.050*	
C19	0.3453 (2)	0.9480 (2)	0.3343 (2)	0.0560 (7)	
H19A	0.3877	0.9881	0.4036	0.067*	
H19B	0.3633	0.8837	0.3602	0.067*	
C20	0.1664 (2)	1.01221 (18)	0.1256 (2)	0.0477 (6)	
H20	0.1135	1.0693	0.1064	0.057*	
C21	0.1057 (2)	0.94262 (17)	0.0232 (2)	0.0470 (6)	
C22	0.1457 (3)	0.8500 (2)	0.0340 (3)	0.0614 (7)	
H22	0.2089	0.8298	0.1064	0.074*	
C23	0.0937 (3)	0.7875 (2)	−0.0602 (4)	0.0788 (10)	
H23	0.1231	0.7262	−0.0516	0.095*	
C24	−0.0014 (3)	0.8161 (3)	−0.1668 (3)	0.0804 (10)	
H24	−0.0371	0.7738	−0.2299	0.096*	
C25	−0.0440 (3)	0.9063 (3)	−0.1805 (3)	0.0721 (9)	
H25	−0.1080	0.9257	−0.2528	0.087*	
C26	0.0093 (2)	0.9688 (2)	−0.0852 (2)	0.0555 (6)	
C27	0.3593 (2)	1.07144 (15)	0.06371 (19)	0.0401 (5)	
C28	0.5037 (2)	1.09515 (16)	0.1552 (2)	0.0401 (5)	
C29	0.6010 (2)	1.09509 (18)	0.0923 (2)	0.0473 (6)	
C30	0.5047 (2)	1.18733 (18)	0.2100 (2)	0.0508 (6)	
C31	0.3687 (2)	0.99588 (17)	−0.0320 (2)	0.0421 (5)	
C32	0.2909 (2)	1.03007 (18)	−0.1534 (2)	0.0446 (6)	
C33	0.2603 (2)	0.9912 (2)	−0.2675 (2)	0.0579 (7)	
H33	0.2888	0.9317	−0.2777	0.070*	
C34	0.1841 (3)	1.0450 (3)	−0.3684 (3)	0.0714 (9)	
H34	0.1621	1.0201	−0.4464	0.086*	
C35	0.1418 (3)	1.1323 (3)	−0.3551 (3)	0.0703 (9)	
H35	0.0929	1.1656	−0.4243	0.084*	
C36	0.1703 (2)	1.1737 (2)	−0.2392 (2)	0.0548 (7)	
C37	0.2469 (2)	1.11967 (17)	−0.1398 (2)	0.0437 (6)	
C38	0.2821 (2)	1.14927 (16)	−0.0176 (2)	0.0440 (6)	
C39	0.2397 (2)	1.23392 (19)	0.0068 (3)	0.0560 (7)	
H39	0.2600	1.2545	0.0868	0.067*	
C40	0.1637 (3)	1.2898 (2)	−0.0929 (3)	0.0665 (8)	
H40	0.1358	1.3481	−0.0771	0.080*	
C41	0.1300 (3)	1.2612 (2)	−0.2111 (3)	0.0641 (8)	
H41	0.0800	1.3000	−0.2738	0.077*	
N1	0.67236 (19)	0.88497 (13)	0.36479 (17)	0.0466 (5)	
N2	0.30451 (17)	1.03727 (14)	0.15349 (17)	0.0429 (4)	
N3	0.5058 (3)	1.25734 (17)	0.2555 (2)	0.0757 (7)	
N4	0.6746 (2)	1.0943 (2)	0.0429 (2)	0.0712 (7)	
O1	0.4578 (2)	0.53771 (14)	0.3864 (2)	0.0816 (7)	
O2	0.8777 (2)	0.88497 (14)	0.52295 (19)	0.0801 (7)	
O3	0.43283 (18)	0.92533 (12)	−0.00412 (16)	0.0568 (5)	
S1	0.16802 (7)	0.96921 (7)	0.27474 (7)	0.0761 (3)	
Cl1	−0.05283 (7)	1.08123 (6)	−0.10849 (7)	0.0774 (2)	

### Atomic displacement parameters (Å^2^)

**Table:**

	*U*^11^	*U*^22^	*U*^33^	*U*^12^	*U*^13^	*U*^23^
C1	0.0724 (18)	0.0524 (18)	0.114 (3)	−0.0146 (15)	0.0384 (19)	−0.0068 (18)
C2	0.0729 (17)	0.0404 (14)	0.0678 (19)	0.0018 (12)	0.0387 (14)	0.0052 (13)
C3	0.122 (3)	0.0527 (17)	0.0529 (17)	−0.0003 (17)	0.0451 (18)	0.0045 (14)
C4	0.096 (2)	0.0501 (16)	0.0446 (15)	0.0006 (15)	0.0291 (14)	−0.0010 (13)
C5	0.0540 (13)	0.0374 (13)	0.0455 (14)	0.0106 (10)	0.0177 (11)	0.0052 (11)
C6	0.0542 (14)	0.0446 (13)	0.0431 (14)	0.0071 (11)	0.0164 (11)	0.0058 (11)
C7	0.0548 (14)	0.0453 (14)	0.0556 (16)	0.0027 (12)	0.0192 (12)	−0.0049 (12)
C8	0.0527 (14)	0.0468 (14)	0.0554 (16)	0.0136 (11)	0.0053 (13)	0.0003 (12)
C9	0.0427 (12)	0.0502 (14)	0.0496 (14)	0.0050 (10)	0.0139 (11)	−0.0034 (11)
C10	0.0378 (11)	0.0432 (13)	0.0453 (13)	0.0026 (10)	0.0075 (10)	−0.0021 (11)
C11	0.0584 (15)	0.0550 (16)	0.0523 (16)	−0.0059 (12)	0.0231 (12)	−0.0023 (12)
C12	0.0711 (18)	0.072 (2)	0.0554 (17)	−0.0046 (15)	0.0238 (15)	−0.0153 (15)
C13	0.0623 (16)	0.0534 (17)	0.074 (2)	−0.0003 (13)	0.0153 (15)	−0.0119 (15)
C14	0.0614 (15)	0.0515 (16)	0.0598 (17)	−0.0057 (12)	0.0150 (13)	0.0022 (13)
C15	0.0498 (14)	0.0565 (16)	0.0464 (14)	−0.0051 (12)	0.0145 (11)	−0.0012 (12)
C16	0.0465 (12)	0.0393 (12)	0.0363 (11)	−0.0007 (10)	0.0144 (10)	0.0009 (10)
C17	0.0450 (12)	0.0396 (12)	0.0330 (11)	0.0011 (9)	0.0137 (10)	0.0003 (9)
C18	0.0463 (12)	0.0432 (12)	0.0349 (11)	−0.0019 (10)	0.0134 (9)	0.0018 (10)
C19	0.0585 (15)	0.0666 (17)	0.0456 (14)	−0.0045 (13)	0.0215 (12)	0.0100 (13)
C20	0.0447 (12)	0.0564 (15)	0.0447 (13)	0.0024 (11)	0.0186 (10)	0.0013 (11)
C21	0.0391 (11)	0.0557 (16)	0.0504 (14)	−0.0065 (10)	0.0207 (10)	0.0008 (12)
C22	0.0550 (15)	0.0594 (17)	0.0718 (19)	−0.0066 (13)	0.0244 (14)	−0.0004 (15)
C23	0.0720 (19)	0.0641 (19)	0.110 (3)	−0.0079 (16)	0.044 (2)	−0.021 (2)
C24	0.0647 (18)	0.096 (3)	0.087 (2)	−0.0124 (19)	0.0346 (18)	−0.036 (2)
C25	0.0526 (15)	0.105 (3)	0.0598 (18)	−0.0043 (16)	0.0212 (14)	−0.0119 (18)
C26	0.0386 (12)	0.0716 (17)	0.0591 (16)	−0.0035 (12)	0.0206 (12)	−0.0023 (14)
C27	0.0419 (11)	0.0411 (12)	0.0367 (12)	0.0019 (10)	0.0127 (9)	0.0049 (10)
C28	0.0419 (11)	0.0400 (12)	0.0375 (11)	−0.0009 (9)	0.0125 (9)	0.0029 (10)
C29	0.0453 (12)	0.0526 (14)	0.0411 (13)	−0.0048 (11)	0.0112 (11)	0.0059 (11)
C30	0.0556 (14)	0.0422 (14)	0.0481 (15)	0.0006 (11)	0.0095 (12)	0.0009 (12)
C31	0.0423 (11)	0.0440 (13)	0.0420 (13)	−0.0021 (10)	0.0169 (10)	0.0003 (10)
C32	0.0366 (11)	0.0579 (15)	0.0387 (13)	−0.0083 (10)	0.0119 (10)	0.0015 (11)
C33	0.0504 (14)	0.0786 (19)	0.0439 (14)	−0.0109 (13)	0.0150 (12)	−0.0078 (14)
C34	0.0656 (18)	0.106 (3)	0.0374 (15)	−0.0122 (18)	0.0112 (13)	−0.0043 (16)
C35	0.0553 (16)	0.103 (3)	0.0413 (16)	−0.0039 (16)	0.0025 (13)	0.0169 (16)
C36	0.0354 (12)	0.0737 (18)	0.0511 (16)	−0.0025 (12)	0.0096 (11)	0.0172 (14)
C37	0.0351 (11)	0.0565 (15)	0.0362 (13)	−0.0027 (10)	0.0079 (9)	0.0069 (11)
C38	0.0394 (11)	0.0470 (14)	0.0446 (14)	−0.0007 (10)	0.0133 (10)	0.0052 (11)
C39	0.0517 (13)	0.0533 (16)	0.0604 (17)	0.0119 (12)	0.0157 (12)	0.0055 (13)
C40	0.0549 (15)	0.0584 (17)	0.081 (2)	0.0139 (13)	0.0165 (15)	0.0128 (16)
C41	0.0448 (14)	0.0725 (19)	0.068 (2)	0.0104 (13)	0.0108 (13)	0.0285 (16)
N1	0.0543 (11)	0.0395 (10)	0.0394 (11)	0.0032 (9)	0.0078 (9)	0.0047 (9)
N2	0.0412 (10)	0.0507 (11)	0.0380 (10)	0.0032 (8)	0.0151 (8)	0.0071 (9)
N3	0.0922 (18)	0.0478 (14)	0.0749 (17)	0.0059 (13)	0.0130 (14)	−0.0066 (13)
N4	0.0615 (14)	0.101 (2)	0.0575 (15)	−0.0105 (14)	0.0292 (12)	0.0029 (14)
O1	0.1154 (17)	0.0505 (12)	0.0911 (17)	−0.0150 (11)	0.0511 (14)	0.0024 (11)
O2	0.0746 (13)	0.0640 (12)	0.0725 (13)	0.0129 (10)	−0.0121 (11)	0.0103 (11)
O3	0.0695 (11)	0.0535 (10)	0.0486 (10)	0.0109 (9)	0.0217 (9)	−0.0015 (8)
S1	0.0604 (4)	0.1237 (7)	0.0530 (4)	−0.0016 (4)	0.0309 (3)	0.0097 (4)
Cl1	0.0530 (4)	0.0809 (5)	0.0861 (5)	0.0059 (4)	0.0085 (4)	0.0137 (4)

### Geometric parameters (Å, º)

**Table:**

C1—O1	1.416 (4)	C19—H19B	0.9700
C1—H1A	0.9600	C20—N2	1.453 (3)
C1—H1B	0.9600	C20—C21	1.522 (4)
C1—H1C	0.9600	C20—S1	1.841 (3)
C2—O1	1.374 (3)	C20—H20	0.9800
C2—C7	1.377 (4)	C21—C26	1.383 (3)
C2—C3	1.381 (4)	C21—C22	1.391 (4)
C3—C4	1.369 (4)	C22—C23	1.381 (4)
C3—H3	0.9300	C22—H22	0.9300
C4—C5	1.382 (4)	C23—C24	1.374 (5)
C4—H4	0.9300	C23—H23	0.9300
C5—C6	1.384 (3)	C24—C25	1.366 (5)
C5—N1	1.419 (3)	C24—H24	0.9300
C6—C7	1.382 (3)	C25—C26	1.391 (4)
C6—H6	0.9300	C25—H25	0.9300
C7—H7	0.9300	C26—Cl1	1.735 (3)
C8—O2	1.204 (3)	C27—N2	1.452 (3)
C8—N1	1.362 (3)	C27—C38	1.517 (3)
C8—C9	1.525 (4)	C27—C31	1.586 (3)
C9—C10	1.502 (3)	C27—C28	1.590 (3)
C9—C16	1.584 (3)	C28—C30	1.470 (4)
C9—H9	0.9800	C28—C29	1.471 (4)
C10—C11	1.387 (4)	C29—N4	1.128 (3)
C10—C15	1.386 (4)	C30—N3	1.136 (3)
C11—C12	1.375 (4)	C31—O3	1.207 (3)
C11—H11	0.9300	C31—C32	1.460 (3)
C12—C13	1.373 (4)	C32—C33	1.375 (3)
C12—H12	0.9300	C32—C37	1.400 (3)
C13—C14	1.363 (4)	C33—C34	1.412 (4)
C13—H13	0.9300	C33—H33	0.9300
C14—C15	1.380 (4)	C34—C35	1.362 (5)
C14—H14	0.9300	C34—H34	0.9300
C15—H15	0.9300	C35—C36	1.411 (4)
C16—N1	1.473 (3)	C35—H35	0.9300
C16—C17	1.510 (3)	C36—C37	1.404 (3)
C16—H16	0.9800	C36—C41	1.405 (4)
C17—C18	1.544 (3)	C37—C38	1.408 (3)
C17—C28	1.593 (3)	C38—C39	1.364 (4)
C17—H17	0.9800	C39—C40	1.419 (4)
C18—N2	1.459 (3)	C39—H39	0.9300
C18—C19	1.524 (3)	C40—C41	1.363 (4)
C18—H18	0.9800	C40—H40	0.9300
C19—S1	1.818 (3)	C41—H41	0.9300
C19—H19A	0.9700		
			
O1—C1—H1A	109.5	C21—C20—S1	112.51 (18)
O1—C1—H1B	109.5	N2—C20—H20	108.2
H1A—C1—H1B	109.5	C21—C20—H20	108.2
O1—C1—H1C	109.5	S1—C20—H20	108.2
H1A—C1—H1C	109.5	C26—C21—C22	117.0 (2)
H1B—C1—H1C	109.5	C26—C21—C20	121.5 (2)
O1—C2—C7	124.3 (3)	C22—C21—C20	121.4 (2)
O1—C2—C3	115.5 (3)	C23—C22—C21	121.5 (3)
C7—C2—C3	120.2 (2)	C23—C22—H22	119.3
C4—C3—C2	120.5 (3)	C21—C22—H22	119.3
C4—C3—H3	119.7	C24—C23—C22	119.8 (3)
C2—C3—H3	119.7	C24—C23—H23	120.1
C3—C4—C5	119.8 (3)	C22—C23—H23	120.1
C3—C4—H4	120.1	C25—C24—C23	120.4 (3)
C5—C4—H4	120.1	C25—C24—H24	119.8
C4—C5—C6	119.8 (2)	C23—C24—H24	119.8
C4—C5—N1	118.8 (2)	C24—C25—C26	119.3 (3)
C6—C5—N1	121.4 (2)	C24—C25—H25	120.3
C7—C6—C5	120.3 (2)	C26—C25—H25	120.3
C7—C6—H6	119.8	C21—C26—C25	121.9 (3)
C5—C6—H6	119.8	C21—C26—Cl1	121.6 (2)
C2—C7—C6	119.4 (2)	C25—C26—Cl1	116.5 (2)
C2—C7—H7	120.3	N2—C27—C38	115.48 (19)
C6—C7—H7	120.3	N2—C27—C31	114.61 (18)
O2—C8—N1	131.6 (3)	C38—C27—C31	102.62 (17)
O2—C8—C9	135.1 (2)	N2—C27—C28	97.68 (15)
N1—C8—C9	93.33 (18)	C38—C27—C28	117.40 (18)
C10—C9—C8	117.5 (2)	C31—C27—C28	109.55 (17)
C10—C9—C16	120.32 (19)	C30—C28—C29	108.5 (2)
C8—C9—C16	84.68 (18)	C30—C28—C27	110.17 (19)
C10—C9—H9	110.7	C29—C28—C27	111.36 (18)
C8—C9—H9	110.7	C30—C28—C17	108.30 (18)
C16—C9—H9	110.7	C29—C28—C17	114.07 (18)
C11—C10—C15	118.3 (2)	C27—C28—C17	104.34 (17)
C11—C10—C9	121.3 (2)	N4—C29—C28	179.1 (3)
C15—C10—C9	120.4 (2)	N3—C30—C28	178.0 (3)
C12—C11—C10	120.6 (3)	O3—C31—C32	129.0 (2)
C12—C11—H11	119.7	O3—C31—C27	123.7 (2)
C10—C11—H11	119.7	C32—C31—C27	107.3 (2)
C13—C12—C11	120.2 (3)	C33—C32—C37	120.4 (2)
C13—C12—H12	119.9	C33—C32—C31	131.9 (2)
C11—C12—H12	119.9	C37—C32—C31	107.7 (2)
C14—C13—C12	119.9 (3)	C32—C33—C34	117.5 (3)
C14—C13—H13	120.0	C32—C33—H33	121.3
C12—C13—H13	120.0	C34—C33—H33	121.3
C13—C14—C15	120.3 (3)	C35—C34—C33	122.1 (3)
C13—C14—H14	119.8	C35—C34—H34	119.0
C15—C14—H14	119.8	C33—C34—H34	119.0
C14—C15—C10	120.6 (3)	C34—C35—C36	121.8 (3)
C14—C15—H15	119.7	C34—C35—H35	119.1
C10—C15—H15	119.7	C36—C35—H35	119.1
N1—C16—C17	113.65 (19)	C37—C36—C41	116.3 (3)
N1—C16—C9	86.81 (16)	C37—C36—C35	115.5 (3)
C17—C16—C9	117.27 (19)	C41—C36—C35	128.1 (3)
N1—C16—H16	112.3	C32—C37—C36	122.7 (2)
C17—C16—H16	112.3	C32—C37—C38	114.1 (2)
C9—C16—H16	112.3	C36—C37—C38	123.1 (2)
C16—C17—C18	116.15 (18)	C39—C38—C37	119.2 (2)
C16—C17—C28	113.05 (18)	C39—C38—C27	132.7 (2)
C18—C17—C28	103.56 (16)	C37—C38—C27	108.0 (2)
C16—C17—H17	107.9	C38—C39—C40	118.2 (3)
C18—C17—H17	107.9	C38—C39—H39	120.9
C28—C17—H17	107.9	C40—C39—H39	120.9
N2—C18—C19	105.65 (18)	C41—C40—C39	122.5 (3)
N2—C18—C17	102.28 (17)	C41—C40—H40	118.8
C19—C18—C17	118.71 (19)	C39—C40—H40	118.8
N2—C18—H18	109.9	C40—C41—C36	120.6 (2)
C19—C18—H18	109.9	C40—C41—H41	119.7
C17—C18—H18	109.9	C36—C41—H41	119.7
C18—C19—S1	106.27 (16)	C8—N1—C5	130.2 (2)
C18—C19—H19A	110.5	C8—N1—C16	95.15 (18)
S1—C19—H19A	110.5	C5—N1—C16	131.75 (18)
C18—C19—H19B	110.5	C27—N2—C20	124.01 (17)
S1—C19—H19B	110.5	C27—N2—C18	108.24 (16)
H19A—C19—H19B	108.7	C20—N2—C18	113.32 (18)
N2—C20—C21	118.0 (2)	C2—O1—C1	117.9 (3)
N2—C20—S1	101.24 (15)	C19—S1—C20	94.89 (12)
			
O1—C2—C3—C4	177.5 (3)	C38—C27—C31—O3	173.6 (2)
C7—C2—C3—C4	−2.0 (5)	C28—C27—C31—O3	48.2 (3)
C2—C3—C4—C5	−0.2 (5)	N2—C27—C31—C32	121.17 (19)
C3—C4—C5—C6	2.0 (4)	C38—C27—C31—C32	−4.8 (2)
C3—C4—C5—N1	−176.7 (3)	C28—C27—C31—C32	−130.26 (18)
C4—C5—C6—C7	−1.7 (4)	O3—C31—C32—C33	5.1 (4)
N1—C5—C6—C7	176.9 (2)	C27—C31—C32—C33	−176.6 (2)
O1—C2—C7—C6	−177.2 (2)	O3—C31—C32—C37	−173.5 (2)
C3—C2—C7—C6	2.3 (4)	C27—C31—C32—C37	4.9 (2)
C5—C6—C7—C2	−0.4 (4)	C37—C32—C33—C34	0.1 (4)
O2—C8—C9—C10	−60.2 (4)	C31—C32—C33—C34	−178.3 (2)
N1—C8—C9—C10	120.3 (2)	C32—C33—C34—C35	0.2 (4)
O2—C8—C9—C16	178.3 (3)	C33—C34—C35—C36	−0.9 (5)
N1—C8—C9—C16	−1.25 (19)	C34—C35—C36—C37	1.2 (4)
C8—C9—C10—C11	−24.6 (3)	C34—C35—C36—C41	−177.7 (3)
C16—C9—C10—C11	75.9 (3)	C33—C32—C37—C36	0.3 (4)
C8—C9—C10—C15	155.8 (2)	C31—C32—C37—C36	179.1 (2)
C16—C9—C10—C15	−103.6 (3)	C33—C32—C37—C38	178.1 (2)
C15—C10—C11—C12	1.4 (4)	C31—C32—C37—C38	−3.1 (3)
C9—C10—C11—C12	−178.2 (2)	C41—C36—C37—C32	178.1 (2)
C10—C11—C12—C13	−0.8 (4)	C35—C36—C37—C32	−0.9 (4)
C11—C12—C13—C14	−0.3 (4)	C41—C36—C37—C38	0.5 (3)
C12—C13—C14—C15	0.8 (4)	C35—C36—C37—C38	−178.6 (2)
C13—C14—C15—C10	−0.3 (4)	C32—C37—C38—C39	−177.2 (2)
C11—C10—C15—C14	−0.8 (3)	C36—C37—C38—C39	0.6 (4)
C9—C10—C15—C14	178.8 (2)	C32—C37—C38—C27	−0.2 (3)
C10—C9—C16—N1	−117.7 (2)	C36—C37—C38—C27	177.6 (2)
C8—C9—C16—N1	1.16 (17)	N2—C27—C38—C39	54.2 (3)
C10—C9—C16—C17	−2.6 (3)	C31—C27—C38—C39	179.6 (3)
C8—C9—C16—C17	116.2 (2)	C28—C27—C38—C39	−60.3 (4)
N1—C16—C17—C18	−58.9 (3)	N2—C27—C38—C37	−122.4 (2)
C9—C16—C17—C18	−157.94 (19)	C31—C27—C38—C37	3.0 (2)
N1—C16—C17—C28	−178.40 (17)	C28—C27—C38—C37	123.2 (2)
C9—C16—C17—C28	82.5 (2)	C37—C38—C39—C40	−1.4 (4)
C16—C17—C18—N2	−144.93 (19)	C27—C38—C39—C40	−177.6 (3)
C28—C17—C18—N2	−20.4 (2)	C38—C39—C40—C41	1.2 (4)
C16—C17—C18—C19	99.3 (3)	C39—C40—C41—C36	−0.1 (4)
C28—C17—C18—C19	−136.1 (2)	C37—C36—C41—C40	−0.7 (4)
N2—C18—C19—S1	27.0 (2)	C35—C36—C41—C40	178.2 (3)
C17—C18—C19—S1	140.95 (18)	O2—C8—N1—C5	−16.2 (5)
N2—C20—C21—C26	−111.8 (3)	C9—C8—N1—C5	163.4 (2)
S1—C20—C21—C26	130.9 (2)	O2—C8—N1—C16	−178.2 (3)
N2—C20—C21—C22	67.5 (3)	C9—C8—N1—C16	1.3 (2)
S1—C20—C21—C22	−49.8 (3)	C4—C5—N1—C8	45.0 (4)
C26—C21—C22—C23	1.4 (4)	C6—C5—N1—C8	−133.6 (3)
C20—C21—C22—C23	−177.9 (3)	C4—C5—N1—C16	−159.3 (3)
C21—C22—C23—C24	−1.2 (5)	C6—C5—N1—C16	22.1 (4)
C22—C23—C24—C25	0.7 (5)	C17—C16—N1—C8	−119.8 (2)
C23—C24—C25—C26	−0.4 (5)	C9—C16—N1—C8	−1.29 (19)
C22—C21—C26—C25	−1.2 (4)	C17—C16—N1—C5	78.7 (3)
C20—C21—C26—C25	178.1 (2)	C9—C16—N1—C5	−162.9 (2)
C22—C21—C26—Cl1	178.01 (19)	C38—C27—N2—C20	48.5 (3)
C20—C21—C26—Cl1	−2.7 (3)	C31—C27—N2—C20	−70.5 (3)
C24—C25—C26—C21	0.7 (4)	C28—C27—N2—C20	173.9 (2)
C24—C25—C26—Cl1	−178.5 (2)	C38—C27—N2—C18	−175.01 (18)
N2—C27—C28—C30	−83.2 (2)	C31—C27—N2—C18	66.0 (2)
C38—C27—C28—C30	40.8 (3)	C28—C27—N2—C18	−49.7 (2)
C31—C27—C28—C30	157.2 (2)	C21—C20—N2—C27	53.6 (3)
N2—C27—C28—C29	156.39 (19)	S1—C20—N2—C27	176.78 (18)
C38—C27—C28—C29	−79.6 (3)	C21—C20—N2—C18	−81.0 (3)
C31—C27—C28—C29	36.8 (2)	S1—C20—N2—C18	42.2 (2)
N2—C27—C28—C17	32.9 (2)	C19—C18—N2—C27	171.04 (18)
C38—C27—C28—C17	156.9 (2)	C17—C18—N2—C27	46.2 (2)
C31—C27—C28—C17	−86.7 (2)	C19—C18—N2—C20	−47.4 (2)
C16—C17—C28—C30	−124.0 (2)	C17—C18—N2—C20	−172.26 (18)
C18—C17—C28—C30	109.5 (2)	C7—C2—O1—C1	6.5 (4)
C16—C17—C28—C29	−3.1 (3)	C3—C2—O1—C1	−173.0 (3)
C18—C17—C28—C29	−129.6 (2)	C18—C19—S1—C20	−4.0 (2)
C16—C17—C28—C27	118.66 (19)	N2—C20—S1—C19	−19.93 (18)
C18—C17—C28—C27	−7.9 (2)	C21—C20—S1—C19	106.93 (19)
N2—C27—C31—O3	−60.4 (3)		

### Hydrogen-bond geometry (Å, º)

Cg1 is the centroid of the C10–C15 ring.

**Table:**

*D*—H···*A*	*D*—H	H···*A*	*D*···*A*	*D*—H···*A*
C18—H18···O3	0.98	2.36	2.961 (2)	119
C20—H20···Cl1	0.98	2.54	3.095 (2)	116
C19—H19*A*···O1^i^	0.97	2.54	3.467 (3)	159
C35—H35···*Cg*1^ii^	0.93	2.83	3.523 (4)	133

Symmetry codes: (i) −*x*+1, *y*+1/2, −*z*+1; (ii) *x*−1, *y*, *z*−1.
